# Involvement of ER stress in apoptosis induced by sialic acid-binding lectin (leczyme) from bullfrog eggs

**DOI:** 10.3892/ijo.2013.2128

**Published:** 2013-10-04

**Authors:** TAKEO TATSUTA, MASAHIRO HOSONO, YUKI MIURA, SHIGEKI SUGAWARA, YUKIKO KARIYA, SENITIROH HAKOMORI, KAZUO NITTA

**Affiliations:** 1 Division of Cell Recognition Study, Institute of Molecular Biomembrane and Glycobiology, Tohoku Pharmaceutical University, Sendai 981-8558;; 2 Fukushima Medical University, Fukushima 960-1295, Japan;; 3 Division of Biomembrane Research, Pacific Northwest Research Institute, Seattle, WA 98122, USA

**Keywords:** lectin, ribonuclease, leczyme, ER-stress, caspase pathway, mitochondria perturbation

## Abstract

Sialic-acid binding lectin (SBL) isolated from bullfrog (*Rana catesbeiana*) oocytes is a multifunctional protein which has lectin activity, ribonuclease activity and cancer-selective antitumor activity. It has been reported that SBL induces apoptosis accompanied by rigid mitochondrial perturbation, which indicates mediation of the intrinsic pathway. However, the mechanism of the antitumor effect of SBL has not been fully elucidated. We report, here, that ER stress is evoked in SBL-treated cells. We show that caspase-4, an initiator caspase of ER stress-mediated apoptosis was activated, and inhibition of caspase-4 resulted in significant attenuation of apoptosis induced by SBL. We analyzed the precise mechanism of activation of the caspase cascade induced by SBL, and found that caspase-9 and -4 are activated upstream of activation of caspase-8. Further study revealed that SBL induces the mitochondrial and ER stress-mediated pathways independently. It is noteworthy that SBL can induce cancer-selective apoptosis by multiple apoptotic signaling pathways, and it can serve as a candidate molecule for anticancer drugs in a novel field.

## Introduction

Chemotherapeutic drugs used in cancer therapy induce apoptosis to tumor cells (1,2). Apoptosis is a physiological form of cell death that plays an important role in normal development, tissue homeostasis and pathological situation (3,4). Two major pathways of apoptosis have been widely recognized, i.e. extrinsic (death receptor; DR) pathway (5,6), and intrinsic (mitochondria mediated) pathway (7,8). More recently, endoplasmic reticulum (ER) stress has drawn attention as the third pathway of apoptosis (9), and has an impact on alternative cell death pathways as potential new targets for cancer therapy.

ER is a multifunctional organelle, and plays roles as an intracellular reservoir of Ca^2+^, and in synthesis of lipid and cholesterol, and synthesis and controlling of the quality of membrane proteins or secreted proteins. A polypeptide translated from mRNA needs to be formatted into a proper higher-order structure to be functional, and this process is called ‘folding’. Folding in ER includes not only formation of a higher-order structure but also glycosylation and formation of disulfide bonds, namely the unique reactions which cannot be seen in folding in the cytoplasm. Correctly folded proteins are transported to Golgi apparatus through vesicular trafficking, where post-translational modifications take place, forming mature proteins. Some proteins that have been misfolded in this process are refolded by ER chaperons such as calnexin and immunoglobulin heavy chain binding protein/glucose regulated protein 78 (Bip/GRP78), or degraded by endoplasmic reticulum-associated degradation (ERAD). Collapse of ER homeostasis induces ER stress derived from the unfolded protein response (UPR), a sequential, pro-survival process for restoring ER functions. UPR is accompanied by the augmentation of folding capacity through increase of molecular chaperon expression and suppression of protein synthesis at transcription or translation level, therefore causing relief of the ER stress by unloading the folding in ER (10–12). The UPR is initiated by activation of three sensors, inositol requiring enzyme 1 (IRE1), activating transcription factor 6 (ATF6), and PKR-like endoplasmic reticulum kinase (PERK). These proteins are transmembrane proteins that monitor accumulation of misfolded proteins in ER lumen, and function as signal transducers of ER stress to cytosol (13). However, in the case of severe prolonged ER stress when UPR and ERAD are not sufficient for the evading the stress, the misfolded proteins are eliminated with whole cells by ER-stress mediated apoptosis. It has been reported that ER stress-dependent apoptosis is mediated mainly by the pro-apoptotic transcription factor CHOP, proapoptotic members of the Bcl-2 family and direct calcium transfer from ER to mitochondria (14,15). Association of ER stress-mediated apoptosis to some pathology such as Alzheimer (16), Parkinson (17), and diabetes (18) has been reported as well as the involvement of the cytotoxic mechanism in the medicinal drugs such as Bortezomib (19) and Nelfinavir (20). It is suggested that targeting ER stress and UPR is a promising strategy for cancer treatment (21).

Sialic acid-binding lectin (SBL) isolated from bullfrog (*Rana catesbeiana*) oocytes was found as a lectin, because SBL agglutinates various kinds of tumor cells and the agglutination was inhibited by sialoglycoprotein or ganglioside (22–24). Agglutination induced by SBL was observed only in tumor cells but not in normal red blood cells and fibroblasts (24). Amino acid sequence of SBL shows that it has homology to the member of RNase A superfamily, and it has been revealed that SBL has pyrimidine base-specific ribonuclease activity (25–28). The antitumor effect of SBL was reported using p388 and L1210 murine leukemia cells *in vitro* and sarcoma 180, Ehrlich and Mep 2 ascites cells *in vivo* (29–31). We have recently reported that SBL shows cytotoxity for various human leukemia cells including MDR cells, and that cytotoxity is induced through caspase-dependent apoptosis in which mitochondrial perturbation occurs as upstream events (32). However, the detail of molecular mechanisms implicated in SBL-induced apoptosis is still unknown. In this study, we investigated the involvement of ER stress in apoptosis triggered by SBL.

## Materials and methods

### Materials

SBL was isolated in sequential chromatography on Sephadex G-75, DEAE-cellulose, hydroxyapatite and SP-Sepharose as described previously (24). Thapsigargin (TG) was purchased from Calbiochem (Darmstadt, Germany). Caspase inhibitors (z-LEVD-fmk, z-VAD-fmk, z-IETD-fmk and z-LEHD-fmk), anti-caspase-4 antibody and anti-caspase-9 antibody were purchased from Medical and Biological Laboratories Co., Ltd (Nagoya, Japan). Anti-caspase-8 antibody and anti-caspase-3 antibody were from Cell Signaling Technology (Beverly, MA, USA). Anti-Bip/GRP78 antibody was from Becton-Dickinson (Franklin Lakes, NJ, USA). Horseradish peroxidase (HRP)-conjugated anti-mouse IgG actibody and HRP-conjugated anti-rabbit IgG andibody were from Zymed (South San Francisco, CA, USA) and Cedarlane Laboratories Ltd (Hornby, ON, Canada), respectively.

### Cell culture

Human leukemia Jurkat T-cells, were obtained from the Cell Resource Center of the Biomedical Research, Institute of Development, Ageing and Cancer, Tohoku University (Sendai, Japan). Cells were routinely kept in RPMI-1640 medium (Nissui Pharmaceutical Co. Ltd., Tokyo, Japan) supplemented with 10% fetal calf serum (FCS), penicillin (100 U/ml) and streptomycin (100 *μ*g/ml) at 37°C in a 95% air and 5% CO_2_ atmosphere.

### Detection of sub G1 population

SBL- and TG-treated cells were harvested, washed and re-suspended in PBS. Then, equal amount of PBS containing Triton X-100 (0.2%), EDTA (4 mM, pH 8.0), RNase A (20 *μ*g/ml), propidium iodide (PI; 40 *μ*g/ml) was added. DNA contents of cells were determined by FACSCalibur (Becton-Dickinson), and the cell population that indicated low DNA contents was counted as sub G1 population.

### Western blot analysis

Whole cell lysate was prepared by lysing the cells with extraction buffer [10 mM Tris-HCl (pH 7.5), 150 mM NaCl, 1% Triton X-100, 5 mM EDTA (pH 8.0), 1 mM phenylmethylsulfonyl fluoride (PMSF) and 1 tablet/10 ml protease inhibitor cocktail (Roche Applied Science, Indianapolis, IN, USA)]. Soluble proteins were collected and concentrations were measured by DC protein assay kit (Bio-Rad, Richmond, CA, USA) in accordance with instructions. Proteins were separated by SDS-PAGE, and transferred to polyviniliden difluoride (PVDF) membrane (GE Healthcare, Little Chalfont, UK). The membrane was blocked by 5% fat-free skim milk for 1 h. After the membrane was washed with TBST [20 mM Tris-HCl (pH 7.6), 137 mM NaCl, 0.05% Tween-20], primary and secondary antibodies were added to the membrane, respectively. The proteins on membrane were detected using ECL western blotting detection reagents (GE Healthcare).

### Detection of x-box binding protein 1 (XBP-1) splicing

Total cellular RNA was isolated from cells using a TRIzol reagent (Invitrogen, Carlsbad, CA, USA). Reverse transcription (RT) was performed using ReverTra Ace (Toyobo, Osaka, Japan) with total RNA (1 *μ*g) and oligo (dT)_12–18_ primers. Splicing of XBP-1 was detected by following the methods of Nakamura *et al* (33). The RT reaction mixture (1 *μ*l) was subjected to PCR for 23 cycles in a final volume of 50 *μ*l of Taq DNA polymerase (1.25 units) (ABgene, Epsom, UK), gene specific forward primer (5′-ACCACAGTCCATGCCATCAC-3′) and reverse primer (5′-TCCACCACCCTGTTGCTG-3′). After initial denaturation at 94°C for 2 min, each of the cycles comprised: at 94°C for 30 sec, at 58°C for 30 sec and at 72°C for 30 sec. To confirm the total expression of XBP-1, PCR products were separated on 1.5% agarose gel, and bands were visualized with ethidium bromide (EtBr) staining. GAPDH expression was also detected as internal control using gene specific forward primer (5′-ACCACAGTCCATGCCATCAC-3′) and reverse primer (5′-TCCACCACCCTGTTGCTGTA-3′). To detect splicing of XBP-1, PCR products were digested with *Apa*LI (10 units) at 37°C for 90 min. Digested sample were separated on 2.5% agarose gel, and bands were visualized with EtBr.

### Treatment of caspase inhibitors

The role of caspase activation in the process of SBL-induce apoptosis was studied by the addition of z-VAD-fmk (pan-caspase inhibitor), z-LEVD-fmk (caspase-4 specific inhibitor), z-IETD-fmk (caspase-8 specific inhibitor) and z-LEHD-fmk (caspase-9 specific inhibitor). Each of the caspase inhibitors [z-LEVD-fmk (2, 10, 30 *μ*M for DNA fragmentation assay, and 30 *μ*M for other assays), z-VAD-fmk, z-IETD-fmk and z-LEHD-fmk (50 *μ*M for all assays)] was added to culture medium 30 min before the addition of SBL or TG.

### Detection of DNA fragmentation

The cells (2×10^5^/ml) were cultured in 100 *μ*l in 96-well plates. After treatment with SBL, the cells were collected by centrifugation, washed with PBS, then lysed with cell lysis buffer [50 mM Tris-HCl (pH 6.8), 10 mM EDTA, 0.5w/v% sodium-N-lauroylsarcosinate]. The samples were incubated for 30 min with RNase A (final concentration: 500 *μ*g/ml) at 50°C, before being digested for 30 min with proteinase K (final concentration: 500 *μ*g/ml) at 50°C. After the samples were electrophoresed on 1.8% agarose gel, DNA bands were visualized by EtBr staining.

### Observation of nuclear morphology

The cells (2×10^5^/ml) were cultured in 5 ml in 6-well plates. After treatment with SBL, the cells were collected by centrifugation and washed with PBS. Then the cells were fixed with 1% paraformaldehyde (100 *μ*l) for 15 min at 4°C, and stained with Hoechst 33258 (50 *μ*l, 1 mg/ml) for 15 min at 4°C. After three washes with PBS, the cells were mounted on slide glass using Prolong gold antifade reagent (Molecular Probes). The fluorescence was visualized with a fluorescence microscope, IX71 microscope (Olympus Corporation, Tokyo, Japan).

### Flow cytometric analysis of Annexin V binding and PI incorporation

Annexin V binding and PI incorporation were detected with a MEBCYTO apoptosis kit (Medical and Biological Laboratories) according to manufacturer’s directions. The cells (2×10^5^/ml) were cultured in 1 ml in 24-well plates. Fluorescence intensity of fluorescein isothiocyanate (FITC)-Annexin V and PI was determined using a FACSCalibur flow cytometer (Becton-Dickinson).

### Reduction of mitochondrial membrane potential (MMP)

MMP was assessed using a fluorescent probe 5, 50, 6, 60-tetra-chloro-1, 10, 3, 30-tetraethylbenzamidazolocarbocyanin iodide (JC-1, AnaSpec, Fremont, CA, USA). Red emission from the dye is attributed to the potential of aggregation of JC-1 in the mitochondria. Green fluorescence reflects the monomeric form of JC-1, appearing in the cytoplasm after mitochondrial membrane depolarization. Cells were cultured in condition of each experiment, and then incubated with JC-1 (2 *μ*M) dye diluted in culture medium at 37°C for 15 min. The cells were washed three times with PBS, and analyzed immediately using FACSCalibur (Becton-Dickinson).

### Statistical analysis

Results were collected from three independent experiments, each performed in triplicate, and data are expressed as mean ± SD. Statistical analysis was performed using GraphPad Prism 3.0 and comparisons were made using one-way or two-way analysis of variance (ANOVA), followed by Bonferroni’s post hoc tests.

## Results

### Time course of apoptotic events in SBL- and TG-treated Jurkat cells

**Figure 1.**
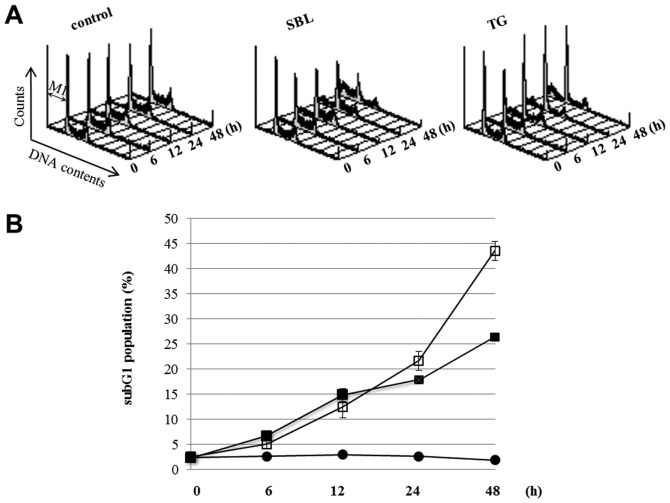
SBL induces time-dependent increment in subG1 population. Jurkat cells were treated with SBL (2 *μ*M) and TG (3 *μ*M) for indicated times. The cells were washed once with PBS, centrifuged and stained with PI. Then, DNA content was measured using FACSCalibur. M1 range represents subG1 population (A), and the percentage is indicated in the line graph (B). Closed circle, open square and closed square represent control, SBL-treated and TG-treated cells, respectively.

**Figure 2.**
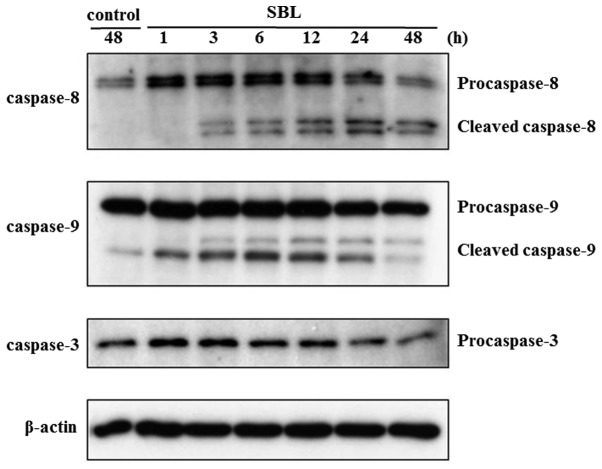
SBL induces time-dependent caspase activation. Jurkat cells were treated with SBL (2 *μ*M) for indicated time. Whole cell lysates were collected, and activation of caspase-8, -9 and -3 was detected by western blot analysis. β-actin was used as loading control.

We have recently shown that SBL possess anti-tumor effect for various leukemia cells including multidrug resistant cells, because SBL executes caspase-dependent apoptosis in which mitochondrial perturbation occurs as upstream events (32). To analyze the detail of the signaling pathway of SBL-induced apoptosis, we first observed the time course of apoptotic events caused by SBL treatment. During apoptosis, it was observed that the cell population indicated low DNA contents resulting from the fragmentation of nucleus and chromatin, and the formation of apoptotic bodies. The sub G1 population described above is considered as an indicator of execution phase of apoptosis. We compared SBL with TG, an endoplasmic reticulum Ca^2+^-ATPase inhibitor, using ER stress inducer as a control. DNA contents of SBL- and TG-treated Jurkat cells were analyzed by flow cytometry, and the sub G1 populations in the cells treating with SBL or TG for 24 and 48 h were 22 and 44% or 18 and 26%, respectively (Fig. 1). Time course of activation of caspases, key proteases in apoptotic process, is also assessed by western blot analysis. As shown in Fig. 2, SBL-induced cleavage of procaspases 9, 8 and 3 was detected from 1, 3 and 24-h treatment, respectively. These results indicate that SBL-induced apoptotic signal is detected from 1-h treatment, as we observed initiator caspase-9 activation.

### Activation of ER stress signaling in SBL-treated Jukat cells

**Figure 3.**
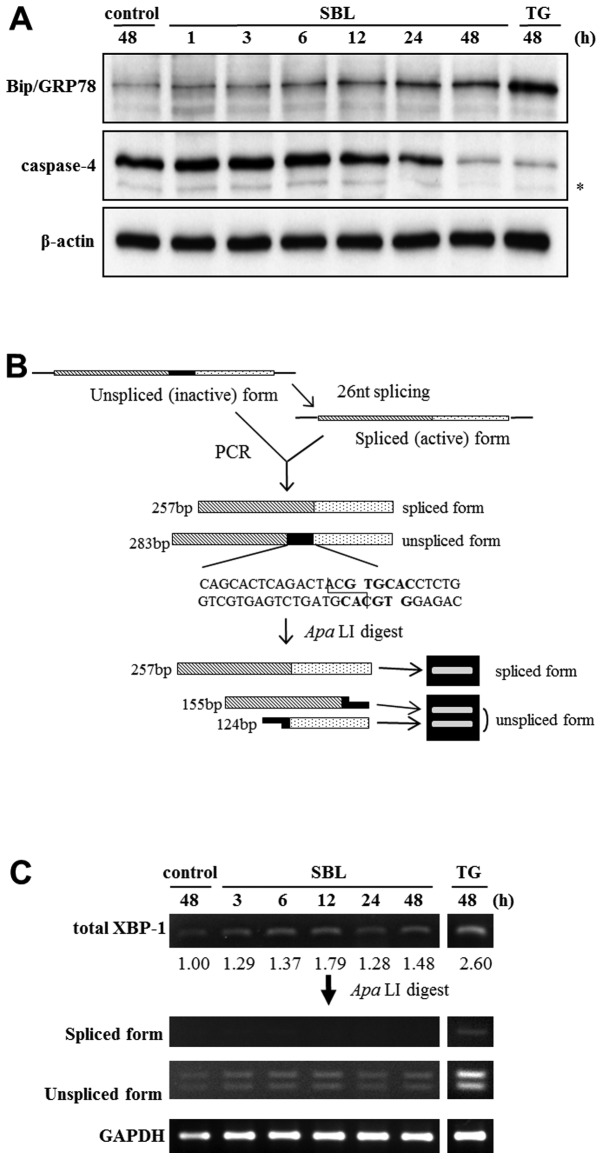
Activation of ER stress signaling in SBL-treated Jurkat cells. (A) Expression of Bip and activation of caspase-4 in SBL-treated Jurkat cells. Jurkat cells were treated with SBL (2 *μ*M) or TG (3 *μ*M) for indicated times. Whole cell lysates were subjected to western blot analysis for detecting Bip/GRP78 and caspase-4. ^*^, non-specific band. (B) Digestion with ApaLI made it easier to discern the expression of spliced and unspliced form of XBP-1. The scheme for distinguishing spliced form from unspliced form of XBP-1 is presented. (C) Expression of XBP-1 in SBL-treated Jurkat cells. Jurkat cells were treated with SBL (2 *μ*M) or TG (3 *μ*M) for indicated times, and RT-PCR was done by use of XBP-1 mRNA. The total expression of XBP-1 was analyzed by 1.5% agarose gel electrophoresis. Products of RT-PCR were subsequently digested with ApaLI for 90 min, to detect inactive or active form derived from XBP-1 DNA. After digestion, the products were electrophoresed using 2.5% agarose gel and stained with EtBr. The total XBP-1 bands were quantified by densitometry, and expressed as a ratio of the intensity of XBP-1 to GAPDH (XBP-1/GAPDH).

To investigate whether SBL induces unfolded protein response (UPR) and ER stress-mediated apoptosis, we assessed the expression of Bip/GRP78 and activation of caspase-4 by western blot analysis, and the elevation of specific splicing of XBP-1 mRNA by RT-PCR followed by subsequent restriction enzyme digestion. Results showed that expression of Bip/GRP78 was elevated after 6 to 48-h of SBL treatment, and that degradation of procaspase-4, namely activation of caspase-4, was detected after 24 to 48-h treatment with SBL (Fig. 3A). It is known that once ER stress was induced, XBP-1 is spliced specifically by IRE1. Because there is an *Apa*LI digestion site on the 27 nt domain on the unspliced form of XBP-1, *Apa*LI digests only unspliced form of XBP-1, and results in smaller two fragments. Digestion with *Apa*LI makes it easier to discern the expression of spliced and unspliced form of XBP-1 (Fig. 3B, upper panel). We found that the spliced and unspliced form of XBP-1 mRNA were detected by RT-PCR followed by subsequent *Apa*LI digestion, and that the total expression of XBP-1 was increased by SBL treatment, but the spliced form of XBP-1 was not increased (Fig. 3B, lower panel).

### Participation of ER stress to SBL-induced apoptosis in Jurkat cells

**Figure 4.**
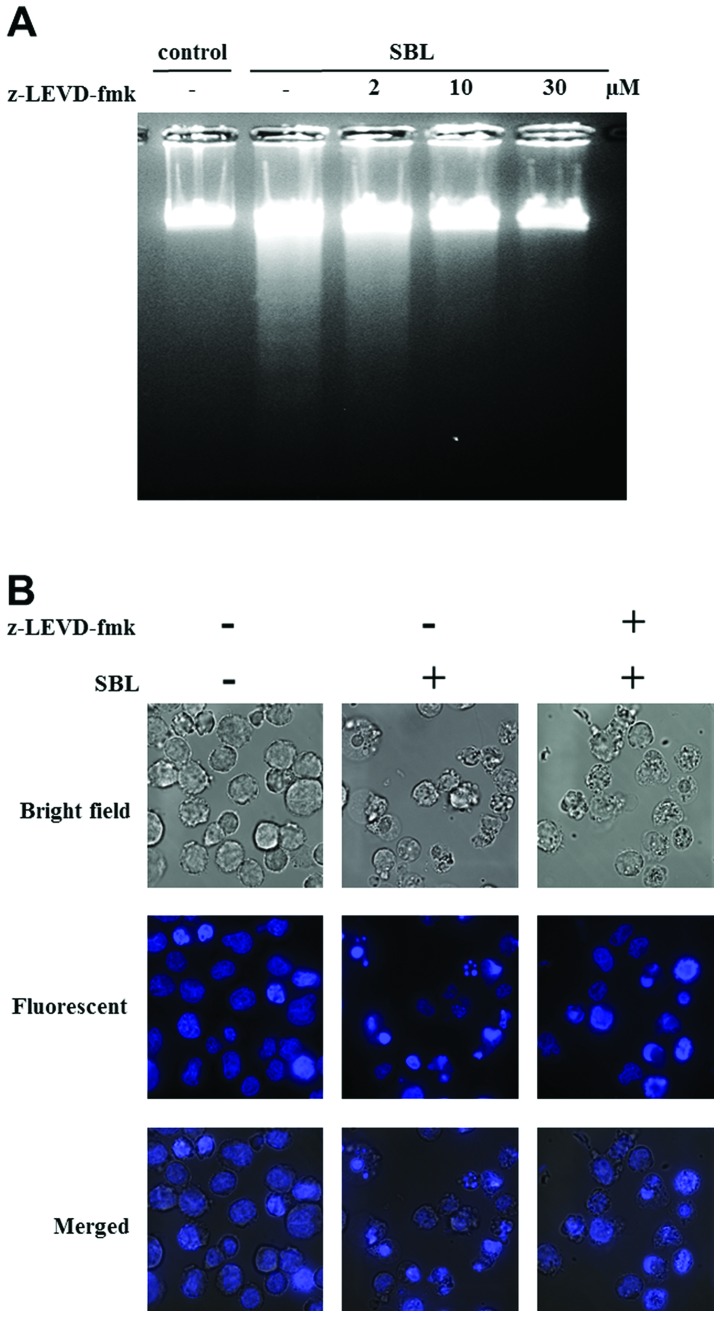

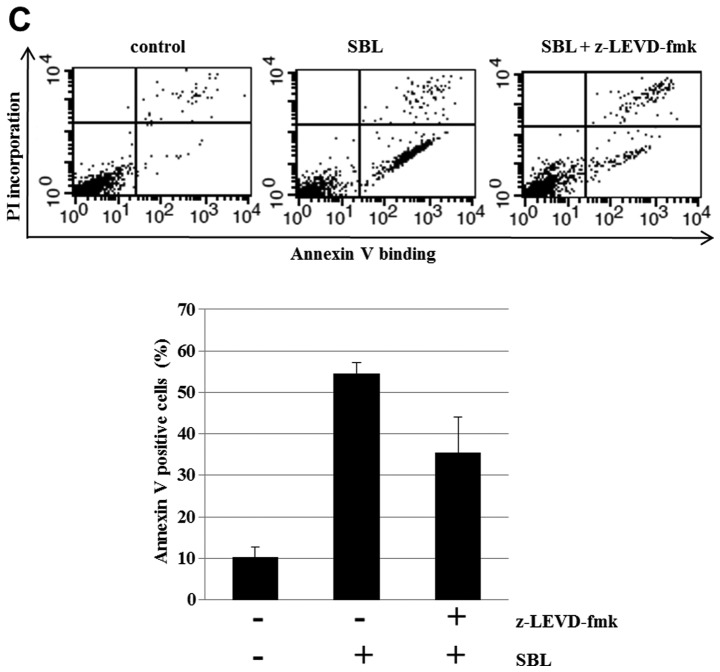
Participation of ER stress to SBL-induced apoptosis in Jurkat cells. (A) Effect of z-LEVD-fmk on SBL-induced DNA fragmentation. Jurkat cells were pretreated with indicated concentrations of z-LEVD-fmk for 30 min, and treated with SBL (2 μM) for 24 h. DNA was prepared from the cells. DNA fragmentation was analyzed by agarose gel electrophoresis, and stained with EtBr. (B) Effect of z-LEVD-fmk on SBL-induced nuclear fragmentation. After pretreatment of Jurkat cells with z-LEVD-fmk (30 μM) for 30 min, the cells were treated with or wihtout SBL (2 μM) for 48 h. Then, the cells were stained with Hoechst 33258, and nuclear fragmentation images were taken by an IX71 microscope with PlanApo 100× 1.45 NA objective. (C) Effect of z-LEVD-fmk on SBL-induced apoptosis. The cells were treated with z-LEVD- fmk (30 μM) for 30 min, and with or without SBL (2 μM) for 24 h. Then, analysis of Annexin V-bound versus PI-incorporated cells was performed by FACScalibur. Percentages of cells divided into lower right-hand (LR) and upper right-hand (UR) quadrant are indicated.

To assess the participation of ER stress signaling to apoptosis induced by SBL, we performed experiments using caspase-4 inhibitor, z-LEVD-fmk. DNA fragmentation induced by SBL was inhibited by z-LEVD-fmk (from 10 *μ*M, in a concentration-dependent manner), and nuclear fragmentation was almost completely inhibited by z-LEVD-fmk at 30 *μ*M (Fig. 4A and B). Staining with Annexin V-PI showed that 54% cells were Annexin V positive in SBL-treated cells, but z-LEVD-fmk-pretreated cells resulted in 20% reduced percentage of Annexin V positive cells (Fig. 4C). These results indicate that caspase-4 activation is involved in SBL-induced apoptosis.

### Comparison of the effects of each caspase inhibitor

**Figure 5.**
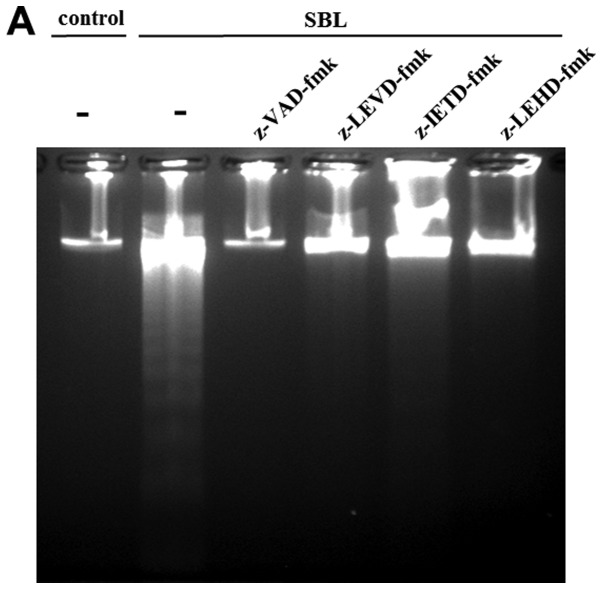

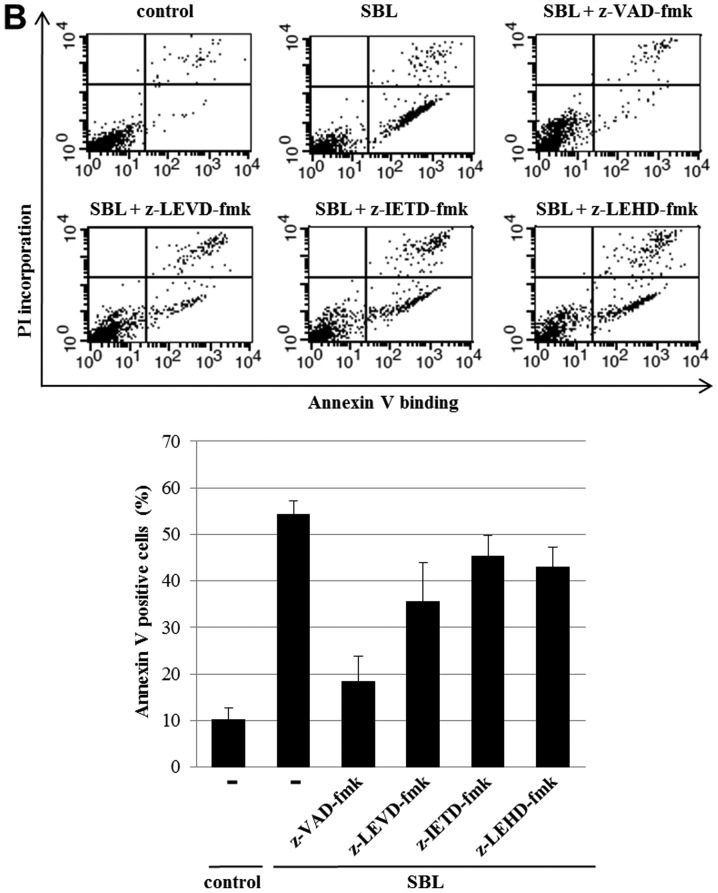
Comparison of effects of four caspase inhibitors. (A) Effect of caspase inhibitors on SBL-induced DNA fragmentation. After pretreatment of Jurkat cells with caspase inhibitors (50 *μ*M for z-VAD-fmk, z-IETD-fmk and z-LEHD-fmk; 30 *μ*M for z-LEVD-fmk) for 30 min, the cells were treated with or without SBL (2 *μ*M) for 24 h. DNA fragmentation was analyzed as described in Fig. 4. (B) Effect of caspase inhibitors on SBL-induced apoptosis. Pretreatment was performed as described in (A), and then the cells were treated with SBL as described in (A). Analysis of Annexin V-binding and PI incorporation was performed as indicated in Fig. 4. Lower panel indicates percentages of cells divided into lower right-hand (LR) and upper right-hand (UR) quadrant, mean ± SE.

Three apoptotic signaling pathways: i) death receptor pathway; ii) mitochondria pathway; and iii) ER stress mediated pathway are well known, and caspase-8, -9 and -4 are considered as the initiator caspase of each pathway, respectively. In SBL-induced apoptotic signal, we detected the activation of caspase-8, -9 and -3 (32), and studied which caspase was activated upstream of apoptotic signal and which caspase is the most important in SBL-induced apoptosis using specific caspase inhibitors. As a result, DNA fragmentation caused by SBL was inhibited completely by pan-caspase inhibitor: z-VAD-fmk; and caspase-4 inhibitor: z-LEVD-fmk. Pretreatment with caspase-9 inhibitor: z-LEHD-fmk also inhibited the DNA fragmentation, but caspase-8 inhibitor, z-IETD-fmk shows relatively low inhibition (Fig. 5A). The effect on induction of apoptosis was also assessed by Annexin V-PI staining, and 36, 19, 9 and 12% inhibition were detected by pretreatment with z-VAD-fmk, z-LEVD-fmk, z-IETD-fmk and z-LEHD-fmk, respectively (Fig. 5B).

**Figure 6.**
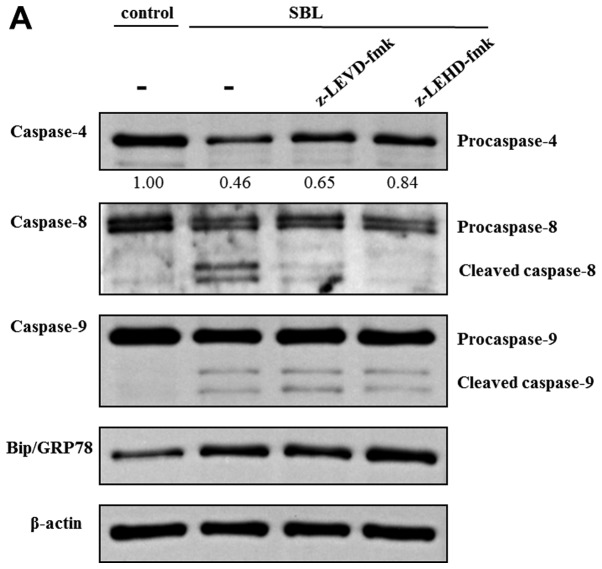

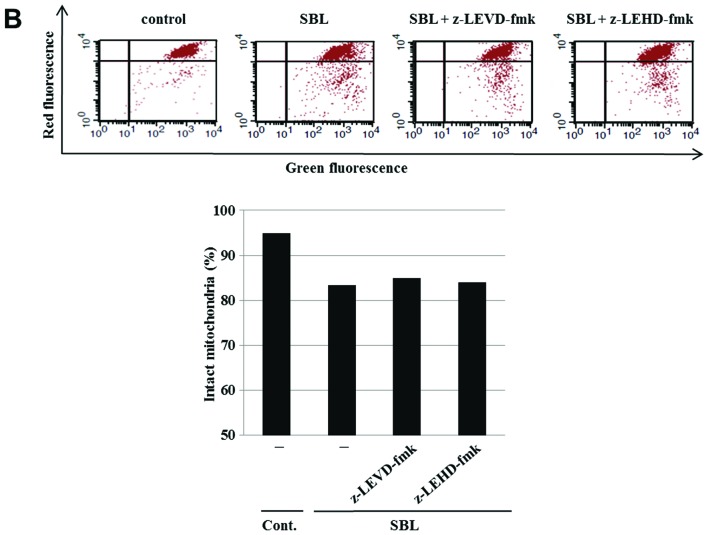
z-LEVD-fmk does not prevent SBL-induced activation of caspase-9 and mitochondrial membrane depolarization. (A) Effect of z-LEHD-fmk and z-LEVD-fmk on SBL-induced caspase activation in Jurkat cells. After pretreatment of Jurkat cells with caspase inhibitors (50 *μ*M for z-LEHD-fmk; 30 *μ*M for z-LEVD-fmk) for 30 min, the cells were treated with or without SBL (2 *μ*M) for 48 h. Whole cell lysates were subjected to western blot analysis using specific antibodies. The caspase-4 bands in the immunoblot were quantified by densitometry and expressed as a ratio of the intensity of caspase-4 to β-actin (caspase-4/actin). (B) Effect of z-LEHD-fmk and z-LEVD-fmk on SBL-induced mitochondrial damage. After pretreatment of Jurkat cells with caspase inhibitors (50 *μ*M for z-LEHD-fmk; 30 *μ*M for z-LEVD-fmk) for 30 min, the cells were treated with SBL (2 *μ*M) for 24 h. Then, cells were stained with JC-1 dye (2 *μ*M) and mitochondrial membrane potential was determined by FACSCalibur. The percentage of cells having intact mitochondrial membrane potential is indicated in the lower pannel.

Because z-LEVD-fmk and z-LEHD-fmk inhibited SBL-induced DNA fragmentation, we assessed the effect of these specific caspase inhibitors on other caspase activation and expression of Bip/FRP78 induced by SBL. In z-LEVD-fmk-pretreated cells, activation of caspase-8 was inhibited, but there was no effect on activation of caspase-9 (Fig. 6A). On the other hand, in z-LEHD-fmk-pretreated cells, activation of caspase-4 as well as that of caspase-8 was diminished. The elevation of Bip/GRP78 expression was not inhibited either by pretreatment with z-LEVD-fmk or z-LEHD-fmk. Previously, we reported that SBL caused rigid mitochondrial perturbation. In the present study, we assessed the effect of caspase-4 and -9 inhibitors on the mitochondrial perturbation induced by SBL. These inhibitors, however, did not inhibit the reduction of MMP triggered by SBL, indicating that mitochondrial perturbation caused by SBL may occur upstream of caspase activation and other events which could be inhibited by caspase inhibitors (Fig. 6B).

## Discussion

We demonstrated that ER stress participated in SBL-induced apoptosis. Disruption of the balance between newly synthesis and quality control mechanism of proteins leads to accumulation of abnormal proteins, i.e. ER stress. The cells try to suppress the stress by elevating the folding clearance though UPR and ERAD. The signal of UPR in eukaryotes can start from each of three transmembrane proteins IRE1, ATF6 and PERK. IRE1 is a type I transmembrane protein activated by dimerization and phosphorylation. Cytosolic domain of IRE1 possesses RNase activity, and activate IRE1 splices XBP-1 mRNA by its RNase activity independently of spliceosome. XBP-1 translated from spliced form of XBP-1 mRNA works as transcription factor, and activates the expression of chaperons such as Bip/GRP78 and factors of ERAD (34–36). ATF6 is a type II transmembrane protein. Once ATF6 detects the accumulation of misfolded proteins, it translocates from ER to golgi through vesicle transport, and is activated by specific cleavage. N-terminal fragment of the cleavage product itself works as a transcription factor, and induces transcription of XBP-1 and molecular chaperones similarly to IRE1 (13). PERK is a type I transmembrane protein which has homology to IRE1, and has kinase activity in its cytosolic domain. PERK is activated by phosphorylation, and the kinase activity causes phosphorylation of the eukaryotic initiation factor 2α (eIF2α), resulting in suppression of global gene expression and unloading protein folding in ER (12). Furthermore, phosphorylation of eIF2α elevates the translation of ATF4, and it is known that ATF4 increases the expression of CHOP and ATF3 (37–40). In the UPR associated factors above, we assessed the expression of Bip/GRP78 and XBP-1 in SBL-treated Jurkat cells to analyze if SBL causes induction of UPR. The results showed that SBL induced elevation of Bip/GRP78 expression, in a time-dependent manner (Fig. 3A). Also, elevated expression of XBP-1 mRNA itself was observed in 48-h treatment with SBL, but the elevation of the active (spliced) form was not observed (Fig. 3C). The elevation of expression of Bip/GRP78 and XBP-1 mRNA suggested that SBL caused the induction of UPR by ER stress, while the fact that there was no elevation of spliced XBP-1 mRNA suggested that the signal transduction may not be mediated by IRE1.

Because the induction of UPR attributed to ER stress was observed in SBL-treated Jurkat cells, we next analyzed whether SBL induces ER stress-mediated apoptosis or not. Caspase-4, a human homolog of mouse caspase-12 is known as an initiator caspase of ER stress-mediated apoptosis (41,42). We revealed that the cleavage of procaspase-4, that is, activation of caspase-4 occurred in SBL-treated Jurkat cells, suggesting that ER stress-mediated apoptosis is involving in SBL-induced apoptosis (Fig. 3).

To assess the participation of ER stress in SBL-induced apoptosis, we performed experiments using caspase-4 specific inhibitor, z-LEVD-fmk. Pretreatment with z-LEVD-fmk diminished SBL-induced DNA fragmentation, dose-dependently (Fig. 4A and B). The percentage of apoptotic cells detected by Annexin V binding assay was also decreased in the cells pretreated with z-LEVD-fmk. Therefore, it is suggested that caspase-4 may play an important role in SBL-induced apopotosis. Caspase-8 and -9, are known as initiator caspases of DR and mitochondrial pathway, respectively, as mentioned above. To examine the contribution of initiator caspases to SBL-induced apoptosis, comparative study was performed with specific caspase inhibitors. We showed that caspase-4 and -9 were prominently involved, because z-LEVD-fmk and z-LEHD-fmk inhibited SBL-induced apoptosis. However, the caspase-8 inhibitor z-IETD was less effective.

**Figure 7.**
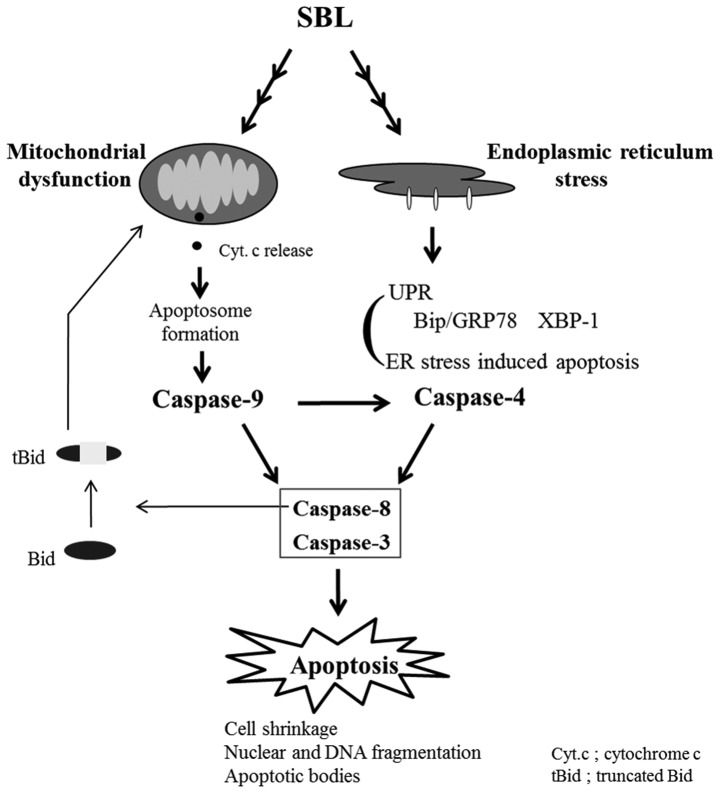
Proposed scheme of various apoptotic pathways involved in SBL-induced apoptosis in Jurkat cells. SBL internalizes into cells, transduces apoptotic signals both in mitochondria and ER. Caspase-9 is activated in mitochondrial pathway, and precedes the apoptotic process. UPR is induced in ER stress pathway, and then caspase-4 is activated. Activation of caspase-9 contributes partially to activation of caspase-4.

Cephalostatin 1 derived from *Cephalodiscus gilchristi* induces ER stress-dependent apoptosis to Jukat cells, and activates caspase-4 causing activation of caspase-9 independently of apoptosome formation. It is reported that caspase-4 is activated upstream of caspase-9 activation in ER stress-dependent apoptosis induced by TG or tunicamycin. Because the clear involvement of caspase-4 and -9 was clarified in SBL-induced apoptosis, we investigated whether SBL-induced apoptotic signal is transduced similarly to the ER stress inducers, by focusing on caspase activation in specific caspase inhibitor-pretreated cells. The results showed the activation of caspase-9 was not affected by the caspase-4 inhibitor z-LEVD-fmk, while activation of caspase-4 was partially diminished by pretreatment with thecaspase-9 inhibitor z-LEHD-fmk (Fig. 6A), indicating that activation of caspase-4 occurs not upstream of caspase-9 activation, but partially dependent on caspase-9 activation. We have recently reported that caspase-8 is activated at downstream of caspase-9 activation in SBL-treated Jurkat cells (32). Taken together, it is suggested that in caspase cascade activated by SBL, caspase-9 is activated as initiator caspase, and it escalates activation of caspase-4, then caspase-8 is activated at downstream of these caspases. Furthermore, activation of caspase-8 is depending on caspase-9 and -4 activation. It is suggested that it participates in the amplification of the apoptotic signal mediated by Bid cleavage. On the other hand, elevated expression of Bip/GRP78 was not affected by z-LEHD-fmk (Fig. 6A), indicating that activation of caspase-9 is not implicating to ER stress induced by SBL. We hypothesized here that mitochondria perturbation and ER stress may occur independently in SBL-treated cells and the activation of caspase-9 is partially involved in activation of caspase-4 (Fig. 7). To confirm the relationship between activation of caspase-4 attributed to ER stress and mitochondrial perturbation, we assessed if the reduction of MMP induced by SBL is affected by z-LEVD-fmk (Fig. 6B), and found that mitochondrial perturbation is neither affected by z-LEVD-fmk nor z-LEHD-fmk. These results support our hypothesis that SBL causes mitochondrial perturbation and ER stress, independently. Furthermore, when we compared the effects of SBL and TG, the cells in sub G1 population were observed in SBL-treated cells more than in TG-treated cells, but ER stress represented by expression of Bip/GRP78 and active form of XBP-1 was observed more rigidly in TG-treated cells (Fig. 1 and 2). These results suggested that SBL causes apoptosis not only by ER stress but also by mitochondrial pathway and mitochondrial pathway may be intensely involved in apoptosis induced by SBL.

We analyzed the signaling mechanism of apoptosis induced by SBL, focusing on induction of ER stress and activation of caspases, and we concluded that SBL can cause multiple apoptotic pathways independently. We have recently reported that SBL activates p38 and JNK MAPKs (32). It has been reported that MAPKs and other molecules such as bcl2-family proteins may participate in ER stress (43,44). The precise antitumor mechanism of SBL and clarification of the relationship between the effects of SBL and the above molecules will advance SBL as a potential candidate for development as an effective anticancer drug.
